# Phenotypic Variation and Carbapenem Resistance Potential in OXA-499-Producing *Acinetobacter pittii*

**DOI:** 10.3389/fmicb.2020.01134

**Published:** 2020-06-09

**Authors:** Linyue Zhang, Ying Fu, Xinhong Han, Qingye Xu, Shanshan Weng, Biyong Yan, Lilin Liu, Xiaoting Hua, Yan Chen, Yunsong Yu

**Affiliations:** ^1^Department of Infectious Diseases, Sir Run Run Shaw Hospital, College of Medicine, Zhejiang University, Hangzhou, China; ^2^Key Laboratory of Microbial Technology and Bioinformatics of Zhejiang Province, Hangzhou, China; ^3^Department of Clinical Laboratory, Sir Run Run Shaw Hospital, College of Medicine, Zhejiang University, Hangzhou, China; ^4^Department of Laboratory Medicine, The Second Affiliated Hospital of Zhejiang University School of Medicine, Hangzhou, China

**Keywords:** *Acinetobacter pittii*, carbapenem resistance, OXA-499, oxacillinase, carbapenemase, phenotypic variation

## Abstract

*Acinetobacter pittii* is increasingly recognized as a clinically important species. Here, we identified a carbapenem-non-resistant *A. pittii* clinical isolate, A1254, harboring *bla*_OXA–__499_, *bla*_OXA–__826_, and *bla*_ADC–__221_. The *bla*_OXA–__499_ genetic environment in A1254 was identical to that of another OXA-499-producing, but carbapenem-resistant, *A. pittii* isolate, YMC2010/8/T346, indicating the existence of phenotypic variation among OXA-499-producing *A. pittii* strains. Under imipenem-selective pressure, the A1254 isolate developed resistance to carbapenems in 60 generations. Two carbapenem-resistant mutants (CAB009 and CAB010) with mutations in the *bla*_OXA–__499_ promoter region were isolated from two independently evolved populations (CAB001 and CAB004). The CAB009 mutant, with a mutation at position −14 (A to G), exhibited a four-fold higher carbapenem minimum inhibitory concentration (MIC) and a 4.53 ± 0.19 log_2_ fold change higher expression level of *bla*_OXA–__499_ than the ancestor strain, A1254. The other mutant, CAB010, with a mutation at position −42 (G to A), showed a two-fold higher carbapenem MIC and a 1.65 ± 0.25 log_2_ fold change higher *bla*_OXA–__499_ expression level than the ancestor strain. The *bla*_OXA–__499_ gene and its promoter region were amplified from the wild-type strain and two mutant isolates and then individually cloned into the pYMAb2-Hyg^*r*^ vector and expressed in *Acinetobacter baumannii* ATCC 17978, *A. pittii* LMG 1035, and *A. pittii* A1254. All the transformed strains were resistant to carbapenem, irrespective of whether they harbored the initial or an evolved promoter sequence, and transformed strains expressing the promoter from the most resistant mutant, CAB009, showed the highest carbapenem MICs, with values of 32–64 μg/ml for imipenem and 128 μg/ml for meropenem. RNA sequencing was performed to confirm the contribution of *bla*_OXA–__499_ to the development of carbapenem resistance. Although the CAB009 and CAB010 transcriptional patterns were different, *bla*_OXA–__499_ was the only differentially expressed gene shared by the two mutants. Our results indicate that carbapenem-non-resistant *Acinetobacter* spp. strains carrying *bla*_OXA_ genes have the potential to develop carbapenem resistance and need to be further investigated and monitored to prevent treatment failure due to the development of resistance.

## Introduction

*Acinetobacter* spp. are increasingly raising serious concern because of their ability to rapidly develop resistance to a wide range of antimicrobials. Among these species, *Acinetobacter baumannii*, *Acinetobacter nosocomialis*, and *Acinetobacter pittii* are the most frequently isolated in hospitals globally (Weber et al., 2015). In the last few decades, relatively few studies have investigated non-*baumannii Acinetobacter* spp., likely owing to their low prevalence and resistant rates (Chen et al., 2019). However, non-*baumannii Acinetobacter* spp. are increasingly being found in clinical specimens and deserve more attention. This is true of *A. pittii*, previously called *Acinetobacter* genomic species three, which is increasingly found in food, clinical patients, and healthy individuals (Yang et al., 2012; Al Atrouni et al., 2016; Silva et al., 2018). Among *Acinetobacter* spp., *A. pittii* is the most commonly identified causative agent of nosocomial infections in patients hospitalized both in general ward and in intensive care units (ICUs) in Germany and is the most prevalent species identified among hospital-acquired *A. calcoaceticus*–*A. baumannii* (ACB) complex bloodstream isolates in France (Schleicher et al., 2013; Pailhories et al., 2018).

The ability of bacteria to rapidly acquire resistance poses crucial challenges to clinical treatment. We have previously shown that *A. baumannii* strains developed greater resistance with within-host evolution, thereby limiting the treatment options (Hua et al., 2017). Similar effects have also been observed for other species. For example, *Pseudomonas aeruginosa* showed rapid and large increases in resistance to carbapenem during antibiotic therapy that were likely due to *de novo* evolution and/or the selection of resistant subpopulations, indicating a potential risk for the rapid spread of antimicrobial resistance (Tueffers et al., 2019). Development of carbapenem resistance in sequential clinical isolates of *Raoultella ornithinolytica* carrying *bla*_OXA–__232_ in a hospitalized patient during the course of ertapenem therapy was recently reported, highlighting the diagnostic challenges posed by strains producing inefficient types of carbapenemase (Iovleva et al., 2019). A more comprehensive understanding of resistance development would provide a molecular basis for improving the treatment of infections. However, to the best of our knowledge, no study to date has investigated the potential for the development of carbapenem resistance in *A. pittii*.

The emergence of carbapenem-resistant *A. pittii* has been reported worldwide. The rate of carbapenem resistance in *A. nosocomialis* and *A. pittii* isolates increased from 7.5% in 2010 to 22% in 2014 (Chen et al., 2019). Carbapenem resistance in *A. pittii* is mainly associated with the production of the carbapenem-hydrolyzing class D β-lactamases (CHDLs), such as OXA-23, OXA-58, OXA-72, OXA-143, and their variants (Zander et al., 2014a; D’Souza et al., 2017; Singkham-In and Chatsuwan, 2018; Chen et al., 2019). Besides, NDM-1-producing *A. pittii* isolates have also been reported in several cases (Yang et al., 2012; Hammerum et al., 2015; Pailhories et al., 2017; Deglmann et al., 2019). The emergence of carbapenem resistance in *A. pittii* reflects its ability to acquire and spread resistance genes, posing a challenge to the management of carbapenem-resistant non-*baumannii Acinetobacter* spp.

In this study, we characterized the *A. pittii* A1254 clinical isolate, which carries *bla*_OXA–__499_, intrinsic *bla*_OXA–__826_, and *bla*_ADC–__221_, but is susceptible to imipenem and intermediate susceptible to meropenem. The carbapenem resistance profile of A1254 was different from that of another OXA-499 producing *A. pittii* strain, YMC2010/8/T346, even though the *bla*_OXA–__499_ genetic environment was identical in the two strains, indicating the existence of phenotypic variation among OXA-499-producing *A. pittii* strains. Thus, we investigated the effect of imipenem exposure in the A1254 isolate to reveal its potential to develop carbapenem resistance.

## Materials and Methods

### Bacterial Isolates and Culture Conditions

*Acinetobacter pittii* A1254 is a clinical strain isolated from the sputum sample of a patient with chronic obstructive pulmonary disease from the People’s Hospital of Quzhou, Zhejiang Province, China, in 2010. The A1254 isolate was initially described in our previous study on the prevalence of carbapenem-hydrolyzing class D β-lactamase genes in *Acinetobacter* spp. isolates (Ji et al., 2014). *A. baumannii* reference strain ATCC 17978, *A. pittii* reference strain LMG1035, and *Escherichia coli* DH5α were employed for cloning experiments. The liquid medium used was Luria–Bertani (LB) broth (Oxford, United Kingdom). The medium was supplemented with imipenem (0.75 μg/ml) as required.

### Antimicrobial Susceptibility Testing

The minimum inhibitory concentrations (MICs) of imipenem and meropenem were evaluated using the broth microdilution method according to the guidelines of the Clinical and Laboratory Standards Institute (CLSI, 2018). Simultaneously, carbapenem MICs against A1254 were evaluated with Etest strips (bioMérieux, Marcy-l’Étoile, France). *E. coli* strain ATCC 25922 was used as quality control. The results were interpreted in accordance with the CLSI breakpoints.

### Experimental Evolution Under Imipenem Selective Pressure

Four single colonies of the *A. pittii* A1254 ancestor strain were inoculated in LB broth supplemented with imipenem (0.75 μg/ml). This concentration was maintained throughout the experimental evolution. All the evolved lineages were passaged daily and independently with shaking (200 rpm) at 37°C. A 20-μl volume of overnight culture was collected and inoculated at a 1:100 dilution daily for 9 days, and the generations were calculated (∼6.64 generations a day) with reference to Nicoloff et al. (2019). The four evolved populations were designated as CAB001, CAB002, CAB003, and CAB004. A different single colony was passaged daily in antibiotic-free LB broth as a blank control.

### Whole-Genome Sequencing and Sequence Analysis

Genomic DNA of the four evolved populations (CAB001–CAB004) was extracted on day 9 using a QIAamp DNA Mini Kit (Qiagen, Valencia, CA, United States) following the manufacturer’s recommendations. The quality and quantity of genomic DNA were determined by agarose gel electrophoresis and a NanoDrop spectrophotometer. A 300-bp library for Illumina paired-end sequencing was constructed from 5 μg of DNA using a Paired-End DNA Sample Prep Kit (Illumina, San Diego, CA, United States). The ancestor strain, *A. pittii* A1254, was sequenced by both long-read nanopore sequencing (Oxford Nanopore Technologies, Oxford, United Kingdom) and Illumina paired-end sequencing. Reads obtained from Illumina paired-end sequencing were used to correct the result of the nanopore sequencing using the Unicycler assembly pipeline (Wick et al., 2017). Paired-end sequence reads were assembled by SPAdes (Bankevich et al., 2012) and the *de novo* assemblies were subsequently annotated using the Prokka pipeline (Seemann, 2014). The genome of A1254 was input to the CGE web server for detection of resistance genes (selected% ID threshold, 90%; selected minimum length, 60%)^1^ (Zankari et al., 2012). Breseq was used to find mutations in evolved populations compared with A1254 (Deatherage and Barrick, 2014). Detected mutations were confirmed by PCR and Sanger sequencing. The primers used are listed in Table 1.

**TABLE 1:** Primers used in the study.

**Name**	**Primer sequence (5′–3′)**	**Target gene/region**	**Use**
499AG F	CTTTCTGCAAACGATGTACT	*bla*_OXA–__499_ upstream region	Verify detected mutations
499AG R	GAGCCTTTTTCAGCAGTT		
499-C-F	tgcggccgcaagcttgtcgacATGAAAAAATTTATACTTCCTATCTTCAGC*	*bla*_OXA–__499_ with (499P-C-F)/without (499-C-F) the upstream region	Recombinant vector construction
499P-C-F	tgcggccgcaagcttgtcgacAAGCTCCATTTAACATAATGGGCG*		
499/P-C-R	cagcaaatgggtcgcggatccTTATATAATCCCTAAATTTTCTAATG*		
826-C-F	tgcggccgcaagcttgtcgacATGACTAAAAAAGCTCTTTTCTTTGC*	*bla*_OXA–__826_ with (826P-C-F)/without (826-C-F) the upstream region	Recombinant vector construction
826P-C-F	tgcggccgcaagcttgtcgacTGACCCAACCCTACCTAA*		
826/P-C-R	cagcaaatgggtcgcggatccCTATAAAATACCGAGTTGTTCCAATCC*		
rpoB-Q-F	TACCTACAAGCGGTTTATCC	*rpoB*	RT-qPCR
rpoB-Q-R	TGTTCGTCATCAAGGTGAAT	*rpoB*	RT-qPCR
499-Q-F	AGCTACAACAACTGAGATTTTC	*bla*_OXA–__499_	RT-qPCR
499-Q-R	CTTGTGTCCCGATGTTCATA	*bla*_OXA–__499_	RT-qPCR
826-Q-F	CATAAAGCAACACCAACTGAA	*bla*_OXA–__826_	RT-qPCR
826-Q-R	AACCAATATCAGCATTACCGA	*bla*_OXA–__826_	RT-qPCR

* Lowercase letters indicate sequences for recombination, capital letters indicate primers for amplification.

### Cloning and Transformation

Fragments of *bla*_OXA–__499_ with or without the promoter region were amplified from the wild-type *A. pittii* A1254 strain and the CAB009 and CAB010 mutants. Additionally, *bla*_OXA–__826_ was also amplified, with or without its promoter region, but only from *A. pittii* A1254. The products were cloned into the *Bam*HI and *Sal*I-digested shuttle vector PYMAb2-Hyg^*r*^ using the ClonExpress^®^ II One Step Cloning Kit (Vazyme Biotech Co., Ltd., Nanjing, China) following the manufacturer’s recommendations. Cloning was performed based on recombination. Briefly, PYMAb2-Hyg^*r*^ was digested with *Bam*HI and *Sal*I. Using the primer pairs shown in Table 1, PCR products comprising the target fragment flanked by the recombination sequences were obtained. Then, the linearized vector, purified PCR product, buffer, and enhanced recombinase (Exnase II) were mixed and incubated for 30 min, yielding the recombinant vectors. The recombinant vectors were transformed into *A. baumannii* ATCC 17978, *A. pittii* LMG1035, and A1254 by electroporation.

### Quantitative Reverse Transcription PCR

Quantitative reverse transcription PCR (RT-qPCR) was performed to measure the expression level of *bla*_OXA–__499_ and *bla*_OXA–__826_ in *A. pittii* A1254 and the CAB009 and CAB010 mutants submitted or not to imipenem selection. Total RNA was extracted using the RNeasy Mini Kit (Qiagen). RNA was reverse transcribed using random hexamers from Invitrogen (Carlsbad, CA, United States) and a reverse transcriptase from Takara Bio (Ôtsu, Japan), according to the manufacturer’s instructions. Quantitative PCR was performed using the SYBR^®^ Premix Ex Taq^TM^ PCR Kit (Takara Bio) in a LightCycler 480 system (Roche Molecular Diagnostics, Rotkreuz, Switzerland). The Ct value of each sample was measured under the following conditions: 95°C for 5 min, followed by 40 amplification cycles at 95°C for 10 s, 52°C for 30 s, and 72°C for 30 s. The *rpoB* gene was used as an internal reference. The primers used are listed in Table 1. Triplicate samples were included in each run, and RT-qPCR was performed three times independently. Data were calculated based on the ΔΔCt method (Livak and Schmittgen, 2001). Log_2_ fold change was used to evaluate the expression levels. Genes were identified as differentially expressed when the |log_2_ fold change| was >1.5 (Wright et al., 2017). Differences in expression levels were assessed by two-tailed Student’s *t*-tests. *P* < 0.05 was considered significant.

### RNA-Sequencing (RNA-Seq)

Three single colonies of each isolate (A1254, CAB009, and CAB010) were grown overnight in LB broth, diluted 1:100 in 100 ml of fresh LB medium, and harvested at the mid-log growth phase. The subsequent RNA extraction, library construction, and transcriptomic analysis were performed by staff at MAGIGENE (Guangzhou, China). RNA was extracted using TRIzol Reagent (Invitrogen) and treated with DNase. rRNA was removed using a Ribo-Zero rRNA Removal Kit (Illumina). Paired-end RNA-sequencing (RNA-Seq) libraries were constructed with the NEBNext^®^ Ultra II^TM^ Directional RNA Library Prep Kit (New England Biolabs, Inc., Ipswich, MA, United States) and sequenced on the Illumina HiSeq/MiSeq NextSeq platform (Illumina). Raw reads were filtered by fastp (version 0.19.7) (Chen et al., 2018). After quality control, the reads were compared with ribosomal RNA (rRNA) sequences in the Rfam database, and unmapped reads were used for subsequent analysis. Filtered reads were mapped to the A1254 genome (GenBank accession number: CP049806-CP049810) using Hisat2 (version 2.1.0) (Kim et al., 2015). The read counts were calculated by RSEM (version 1.3.1) (Li and Dewey, 2011). The output data were analyzed by edgeR (version 3.20.2) (Robinson et al., 2010) and differences in the expression profiles of CAB009 and CAB010 were assessed by the expression ratio of each gene between the mutant and A1254. Genes were considered to be differentially expressed if their false discovery rate (FDR) was <0.05 and the |log_2_ fold change| was >1.5.

## Results

### Resistance Genes and the Genetic Environment of *bla*_OXA–__499_ in *A. pittii* A1254

Based on CLSI guidelines, *A. pittii* A1254 was susceptible to imipenem (MIC, 2 μg/ml) and intermediate susceptible to meropenem (MIC, 4 μg/ml) using the broth microdilution method. However, when determined by Etest strips, A1254 was susceptible to both imipenem (MIC, 1.5 μg/ml) and meropenem (MIC, 2 μg/ml), consistent with the result reported for when A1254 was first described (Ji et al., 2014). Although there was a one-fold difference, the MIC of meropenem evaluated by the two methods was the breakpoint for susceptibility and intermediate susceptibility, respectively. Because in this study we also employed the broth microdilution method to determine the MICs for the other strains, we adopted 4 μg/ml as the meropenem MIC against A1254 and determined the strain to be intermediate susceptible to meropenem.

**FIGURE 1:**
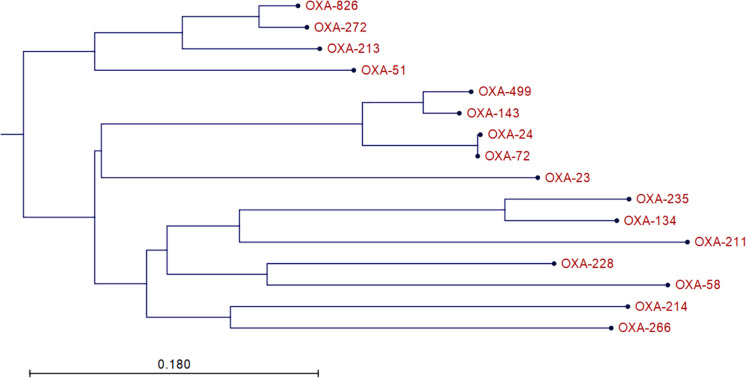
Phylogenetic tree of relative OXA variants. The neighbor-joining tree was generated using CLC Main Workbench 8.5.1 (QIAGEN Bioinformatics).

A1254 carries four plasmids in addition to its 4,065,905-bp-long chromosome. We identified two oxacillinase (OXA) genes in A1254: *bla*_OXA–__499_ and a variant of *bla*_OXA–__500_. *bla*_OXA–__499_ is a variant of *bla*_OXA–__143_ identified in 2017 and is reported to confer resistance to carbapenem (D’Souza et al., 2017). The *bla*_OXA–__500_ variant was submitted to National Center for Biotechnology information (NCBI) as a member of the OXA-213 family, and NCBI designated it as *bla*_OXA–__826_. The phylogenetic tree of relative OXA variants is shown in Figure 1. However, according to a recent study, it might be more appropriate to classify OXA-826 into the OXA-272-like family, which may be the intrinsic *A. pittii* OXA (Kamolvit et al., 2014; D’Souza et al., 2017). We also found and submitted a variant of *bla*_ADC–__25_, which was designated as *bla*_ADC–__221_ by NCBI. *bla*_OXA–__499_, *bla*_OXA–__826_, and *bla*_ADC–__221_ are all located on the chromosome. No other resistance genes were detected in A1254.

We compared the genetic environment of *bla*_OXA–__499_ in A1254 with that in the *A. pittii* isolate YMC2010/8/T346 in which *bla*_OXA–__499_ was first identified (GenBank accession number: CP017938). In the YMC2010/8/T346 isolate, *bla*_OXA–__499_ is located within a 4,085-bp genomic fragment insertion consisting of a putative peptidase gene, *bla*_OXA–__499_, and a TonB-dependent receptor plug domain, along with Xer C/D like recombination sites (D’Souza et al., 2017). The same fragment was identified in A1254 (100% identity).

### Carbapenem-Resistant Evolved Populations and Mutations

We serially passaged four populations independently for 9 days (∼6.64 generations per day). On day 9 (∼60 generations), we found that two of the four populations (CAB001 and CAB004) had become resistant to both imipenem and meropenem. For population 1, CAB001, the imipenem MIC increased to 32 μg/ml (a four-fold increase) and that of meropenem increased to 64 μg/ml (also a four-fold increase). For population 4, CAB004, the imipenem MIC increased to 8 μg/ml (a two-fold increase) while that of meropenem increased to 32 μg/ml (a three-fold increase).

To identify the mutations responsible for the increased MICs (at least two-fold) against CAB001 and CAB004, we compared the genome of each evolved population to the genome sequence of the A1254 ancestor strain. Mutations with a frequency >70% are listed in Supplementary Table S1. Mutations occurring in population two (CAB002) and population three (CAB003) where the imipenem MIC showed only a one-fold increase (4 μg/ml) were excluded from further analysis. Therefore, the mutation in the promoter region of *bla*_OXA–__499_, with a frequency of 100%, was the only mutation in CAB001 remaining for further analysis. A colony from the CAB001 population harboring this mutation was isolated and designated as CAB009.

Based on the evidence from CAB001, we suspected that the mutation in the promoter region of *bla*_OXA–__499_ might be responsible for the increased carbapenem MICs in the population. To explore whether this mutation also existed in other populations, we isolated single colonies from CAB002–CAB004 and analyzed the sequence of the *bla*_OXA–__499_ promoter region in each colony by PCR and Sanger sequencing. We identified another mutation in the promoter region of *bla*_OXA–__499_ in a CAB004-derived colony, and we designated this mutant as CAB010. No mutations were found in the promoter region of *bla*_OXA–__499_ in CAB002- and CAB003-derived colonies. Carbapenem MICs against the ancestor strain and the mutants are listed in Table 2.

**TABLE 2:** Minimum inhibitory concentrations (MICs) for strains and transformants harboring recombinant vectors.

	**MIC (μg/mL)**	
**Strain and (recombinant) plasmid**	**Imipenem**	**Mero- penem**
*Acinetobacter pittii* A1254	2	4
*A. pittii* CAB009	32	64
*A. pittii* CAB010	8	16
*A. pittii* A1254 + pYMAb2_Hyg^*r*^	2	4
*A. pittii* A1254 + pYMAb2_Hyg^*r*^:OXA499	2	4
*A. pittii* A1254 + pYMAb2_Hyg^*r*^:OXA499_P^*a*^	16	64
*A. pittii* A1254 + pYMAb2_Hyg^*r*^:OXA499_P009^*b*^	32	128
*A. pittii* A1254 + pYMAb2_Hyg^*r*^:OXA499_P010^*c*^	16	64
*A. pittii* A1254 + pYMAb2_Hyg^*r*^:OXA826	2	8
*A. pittii* A1254 + pYMAb2_Hyg^*r*^:OXA826_P^*d*^	2	8
*Acinetobacter baumannii* ATCC 17978	0.125	0.25
*A. baumannii* ATCC 17978 + pYMAb2_Hyg^*r*^	0.125	0.5
*A. baumannii* ATCC 17978 + pYMAb2_Hyg^*r*^:OXA499	0.125	0.5
*A. baumannii* ATCC 17978 + pYMAb2_Hyg^*r*^:OXA499_P	16	128
*A. baumannii* ATCC 17978 + pYMAb2_Hyg^*r*^:OXA499_P009	32	128
*A. baumannii* ATCC 17978 + pYMAb2_Hyg^*r*^:OXA499_P010	32	128
*A. baumannii* ATCC 17978 + pYMAb2_Hyg^*r*^:OXA826	0.25	0.25
*A. baumannii* ATCC 17978 + pYMAb2_Hyg^*r*^:OXA826_P	0.25	0.25
*A. pittii* LMG 1035	0.06	0.25
*A. pittii* LMG 1035 + pYMAb2_Hyg^*r*^	0.125	0.125
*A. pittii* LMG 1035 + pYMAb2_Hyg^*r*^:OXA499	0.125	0.25
*A. pittii* LMG 1035 + pYMAb2_Hyg^*r*^:OXA499_P	16	64
*A. pittii* LMG 1035 + pYMAb2_Hyg^*r*^:OXA499_P009	64	128
*A. pittii* LMG 1035 + pYMAb2_Hyg^*r*^:OXA499_P010	16	64
*A. pittii* LMG 1035 + pYMAb2_Hyg^*r*^:OXA826	0.125	0.25
*A. pittii* LMG 1035 + pYMAb2_Hyg^*r*^:OXA826_P	0.125	0.25

*^*a*^OXA499_P indicates *bla*_*OXA–*__*499*_ with its natural promoter cloned from A1254. ^*b*^OXA499_P009 indicates *bla*_*OXA–*__*499*_ with its mutated promoter cloned from CAB009. ^*c*^OXA499_P010 indicates *bla*_*OXA–*__*499*_ with its mutated promoter cloned from CAB010. ^*d*^OXA826_P indicates *bla*_*OXA–*__*826*_ with its natural promoter cloned from A1254.*

**FIGURE 2:**
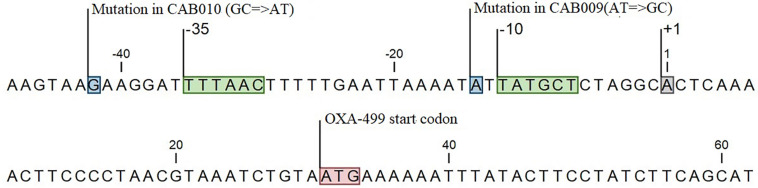
Locations of mutations in the carbapenem-resistant mutants CAB009 and CAB010 (boxed in blue). The start codon (boxed in red), the transcription initiation site (boxed in gray), and the −10 and −35 regions (boxed in green) are also indicated.

Thus, we identified two mutations in the promoter region of *bla*_OXA–__499_ in CAB009 and CAB010 that might mediate carbapenem resistance. The promoter region of *bla*_OXA–__499_ was defined based on a previous study (Zander et al., 2014a). In the *A. pittii* CAB009 mutant from population one, we identified an A to G base substitution located one base upstream of the −10 region (position −14) (Figure 2), yielding a 5′-TG-3′ motif in the extended −10 element. In the *A. pittii* CAB010 mutant from population 4, we identified a G to A base substitution upstream of the −35 hexamer (position −42) within the upstream (UP) element (Figure 2).

### Relative Expression Levels of *bla*_OXA–__499_ and *bla*_OXA–__826_ in the Mutants

**FIGURE 3:**
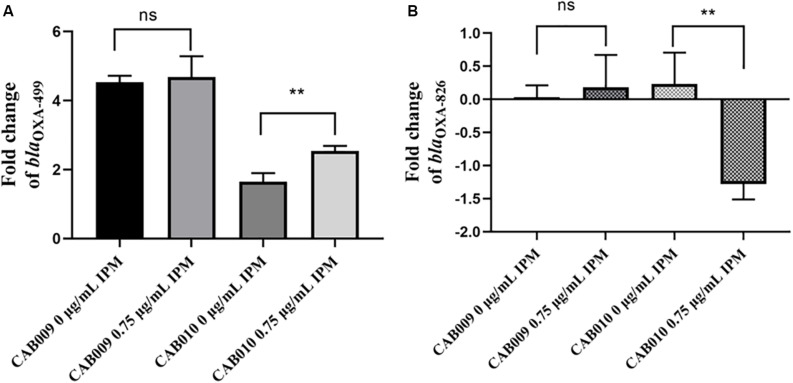
Log_2_ fold changes of **(A)** *bla*_OXA–__499_ and **(B)** *bla*_OXA–__826_ expression levels in mutants undergoing or not imipenem selection pressure. RT-qPCR analysis was performed with three biological and technical replicates per experiment. The 2^–ΔΔCt^ method was applied to calculate the fold change using *rpoB* as the reference gene and *Acinetobacter pittii* A1254 as the reference strain. The expression level was calculated as the log_2_ fold change. The bars represent means ± SD from triplicate biological repeats. The *P*-values (each mutant undergoing or not imipenem selection pressure) were determined by two-tailed Student’s *t*-tests. **P < 0.008; ns, not significant. IPM, imipenem.

We next performed RT-qPCR to determine how the mutations affected the expression levels of *bla*_OXA–__499_ and *bla*_OXA–__826_. The results revealed that the expression level of *bla*_OXA–__499_ increased significantly in CAB009, both in antibiotic-free LB broth (4.53 ± 0.19) and in LB broth supplemented with 0.75 μg/ml imipenem (4.68 ± 0.60). However, the difference between the two conditions was not significant (*P* = 0.6931) (Figure 3A). In CAB010, a more significant increase in *bla*_OXA–__499_ expression was observed under 0.75 μg/ml imipenem pressure (2.54 ± 0.15) compared with that in antibiotic-free LB broth (1.65 ± 0.25) (*P* = 0.006) (Figure 3A). The higher expression level of *bla*_OXA–__499_ in CAB009 might explain why it was more resistant to carbapenem than CAB010 (Table 2). Under imipenem selection pressure, there was a significant decrease in the expression level of *bla*_OXA–__826_ in CAB010 but not CAB009 (Figure 3B). However, based on the |log_2_ fold-change| threshold (>1.5), only *bla*_OXA–__499_ was identified as being differentially expressed in both CAB009 and CAB010. The RT-qPCR results indicated that *bla*_OXA–__499_, but not *bla*_OXA–__826_, played an important role in the development of carbapenem resistance. Supplementary Table S2 depicts the fold changes normalized by *rpoB* and *bla*_OXA–__826_, respectively.

### The Results of Cloning and Transformation

To examine how the mutations affect carbapenem resistance, we amplified a sequence containing either *bla*_OXA–__499_ alone or *bla*_OXA–__499_ together with an upstream 114-bp segment that included the promoter region, from A1254, CAB009, and CAB010. The sequence of *bla*_OXA–__826_ with or without an upstream segment (446 bp in total, comprising a putative promoter region) was amplified from A1254. All the amplified products were cloned into the pYMAb2-Hyg^*r*^ vector. The recombinant vectors were electroporated into *A. baumannii* ATCC 17978, *A. pittii* LMG 1035, and *A. pittii* A1254 for expression analysis. The strains carrying the corresponding recombinant vectors and their MICs are listed in Table 2.

The results were similar among the transformed strains with different genetic backgrounds. *bla*_OXA–__499_ could not confer resistance to carbapenem without the upstream sequence. However, all the recombinant strains harboring *bla*_OXA–__499_ with its original promoter became resistant to imipenem (MIC, 16 μg/ml) and meropenem (MIC, 64–128 μg/ml). Similar MICs were observed for recombinant strains harboring *bla*_OXA–__499_ containing the promoter from CAB010 (P010), except for OXA-499_P010-transformed *A. baumannii* ATCC 17978, showing a one-fold higher imipenem MIC (32 μg/ml) than OXA-499_P-transformed strains (16 μg/ml). Among the *A. baumannii* and *A. pittii* strains, *bla*_OXA–__499_ containing the CAB009 promoter (P009) conferred the highest imipenem MICs (32–64 μg/ml, one- to two-fold change) when compared with the recombinant strains harboring the original promoter. Increased meropenem MICs were only observed for *A. pittii* A1254 and LMG 1035 (128 μg/ml, one-fold).

### Transcriptomic Analysis

Differentially expressed genes between A1254 and CAB009 or CAB010 were identified based on the defined threshold. Overall, although the transcriptional patterns of CAB009 and CAB010 were different, *bla*_OXA–__499_ was the only differentially expressed gene shared by the two mutants.

Eighty-eight differentially expressed genes were identified in CAB009 (Table 3). Among them, 82 were upregulated and six downregulated. A stress-induced protein-encoding gene G8E09_11565 showed a 5.22 log_2_ fold change higher expression level in CAB009 than in A1254, ranking first in the expression profile. This was followed by *bla*_OXA–__499_, which showed a 4.70 log_2_ fold change higher expression level. We only identified three genes that were differentially expressed between CAB010 and A1254 (Table 4). The expression of *bla*_OXA–__499_ was 2.04 log_2_ fold change higher in CAB010 than in A1254. G8E09_06800 (encoding a LysR family transcriptional regulator) and G8E09_06805 (encoding a type one glutamine amidotransferase domain-containing protein) showed 1.73 and 1.62 log_2_ fold change higher expression levels than A1254, respectively.

**TABLE 3:** Differentially expressed genes in CAB009 compared with A1254.

**Gene_ID**	**Description**	**log_2_ FC^*a*^**	***P*-value**	**FDR**
G8E09_11565	Stress-induced protein	5.22	0.0000	0.0004
G8E09_16100	OXA-143 family carbapenem-hydrolyzing class D beta-lactamase OXA-499	4.70	0.0000	0.0000
G8E09_11570	Hypothetical protein	3.78	0.0000	0.0000
G8E09_11590	Damage-inducible protein CinA	3.59	0.0000	0.0001
G8E09_08710	Muconolactone Delta-isomerase	3.37	0.0000	0.0000
G8E09_09185	Hypothetical protein	3.27	0.0002	0.0095
G8E09_12900	Benzoate 1,2-dioxygenase small subunit	3.21	0.0000	0.0000
G8E09_12895	Benzoate 1,2-dioxygenase large subunit	3.08	0.0000	0.0000
G8E09_10220	Hypothetical protein	3.05	0.0000	0.0000
G8E09_07105	Hypothetical protein	3.04	0.0001	0.0043
G8E09_12625	DUF4142 domain-containing protein	3.00	0.0000	0.0000
G8E09_09755	Hypothetical protein	2.94	0.0000	0.0001
G8E09_11585	Iron-containing redox enzyme family protein	2.92	0.0001	0.0047
G8E09_03990	BapA prefix-like domain-containing protein	2.85	0.0000	0.0000
G8E09_14955	Trehalose-phosphatase	2.82	0.0000	0.0000
G8E09_08715	Muconate cycloisomerase	2.69	0.0000	0.0000
G8E09_11610	Hypothetical protein	2.68	0.0000	0.0000
G8E09_09795	Hypothetical protein	2.66	0.0000	0.0003
G8E09_08150	SOS response-associated peptidase	2.66	0.0000	0.0000
G8E09_12315	Hypothetical protein	2.63	0.0000	0.0011
G8E09_08605	MFS transporter	2.60	0.0000	0.0016
G8E09_07245	DNA breaking-rejoining protein	2.58	0.0000	0.0004
G8E09_12905	Ring-hydroxylating dioxygenase ferredoxin reductase family protein	2.49	0.0000	0.0000
G8E09_10205	Hypothetical protein	2.45	0.0022	0.0488
G8E09_08615	Amidase	2.45	0.0010	0.0278
G8E09_08625	IacB protein	2.40	0.0001	0.0048
G8E09_14200	Hypothetical protein	2.39	0.0001	0.0044
G8E09_08105	Hypothetical protein	2.39	0.0001	0.0061
G8E09_08620	Acyl-CoA dehydrogenase	2.39	0.0004	0.0138
G8E09_08600	OprD family porin	2.37	0.0000	0.0000
G8E09_11790	Acyl-CoA dehydrogenase	2.35	0.0000	0.0013
G8E09_14960	Trehalose-6-phosphate synthase	2.33	0.0012	0.0314
G8E09_12910	1,6-dihydroxycyclohexa-2,4-diene-1-carboxylatedehydrogenase	2.25	0.0000	0.0009
G8E09_10090	2-oxo acid dehydrogenase subunit E2	2.24	0.0000	0.0012
G8E09_07160	Type 1 glutamine amidotransferase	2.23	0.0001	0.0076
G8E09_14205	Non-heme iron oxygenase ferredoxin subunit	2.22	0.0004	0.0151
G8E09_08630	Nuclear transport factor 2 family protein	2.22	0.0002	0.0092
G8E09_09095	Type 1 glutamine amidotransferase domain-containing protein	2.19	0.0000	0.0003
G8E09_08650	Oxidoreductase	2.15	0.0000	0.0037
G8E09_12920	Aromatic acid/H + symport family MFS transporter	2.15	0.0000	0.0000
G8E09_08635	Aromatic ring-hydroxylating dioxygenase subunit alpha	2.12	0.0001	0.0073
G8E09_10085	Dihydrolipoyl dehydrogenase	2.12	0.0000	0.0018
G8E09_11775	Enoyl-CoA hydratase	2.10	0.0006	0.0196
G8E09_08705	Catechol 1,2-dioxygenase	2.10	0.0000	0.0008
G8E09_00265	DUF1328 domain-containing protein	2.08	0.0004	0.0151
G8E09_09085	NAD(P)H-binding protein	2.06	0.0000	0.0000
G8E09_08640	Hypothetical protein	2.02	0.0019	0.0437
G8E09_10100	Thiamine pyrophosphate-dependent dehydrogenaseE1 component subunit alpha	2.02	0.0001	0.0064
G8E09_10095	Alpha-ketoacid dehydrogenase subunit beta	2.02	0.0001	0.0065
G8E09_14195	Aromatic ring-hydroxylating dioxygenase subunit alpha	1.97	0.0007	0.0205
G8E09_08645	SDR family oxidoreductase	1.94	0.0002	0.0089
G8E09_03110	Peroxiredoxin	1.93	0.0000	0.0003
G8E09_07120	Molecular chaperone	1.91	0.0000	0.0014
G8E09_07115	Spore coat protein U domain-containing protein	1.90	0.0006	0.0189
G8E09_07200	Minor capsid protein	1.88	0.0016	0.0387
G8E09_07955	Hypothetical protein	1.85	0.0000	0.0004
G8E09_17600	Serine hydrolase family protein	1.83	0.0012	0.0314
G8E09_11605	Hypothetical protein	1.81	0.0005	0.0182
G8E09_12810	Hypothetical protein	1.79	0.0006	0.0198
G8E09_04225	Transglycosylase SLT domain-containing protein	1.79	0.0008	0.0229
G8E09_09750	Hypothetical protein	1.76	0.0000	0.0024
G8E09_14055	Hypothetical protein	1.76	0.0002	0.0089
G8E09_06975	Hypothetical protein	1.76	0.0000	0.0014
G8E09_11740	Enoyl-CoA hydratase	1.76	0.0003	0.0124
G8E09_12805	LysE family transporter	1.75	0.0000	0.0004
G8E09_08700	3-oxoacid CoA-transferase subunit A	1.74	0.0001	0.0044
G8E09_11745	SDR family oxidoreductase	1.74	0.0012	0.0318
G8E09_12925	OprD family porin	1.73	0.0001	0.0064
G8E09_10340	Hypothetical protein	1.72	0.0001	0.0067
G8E09_19180	GlsB/YeaQ/YmgE family stress response membrane protein	1.71	0.0020	0.0463
G8E09_01795	Hemerythrin domain-containing protein	1.71	0.0008	0.0244
G8E09_12835	Heavy-metal-associated domain-containing protein	-1.70	0.0002	0.0091
G8E09_07185	Hypothetical protein	1.67	0.0008	0.0229
G8E09_18175	Flavodoxin family protein	-1.65	0.0001	0.0044
G8E09_12485	Hypothetical protein	1.64	0.0000	0.0033
G8E09_08655	Flavin reductase family protein	1.63	0.0000	0.0017
G8E09_09715	Fimbria/pilus periplasmic chaperone	-1.61	0.0000	0.0012
G8E09_12830	Copper-translocating P-type ATPase	-1.59	0.0001	0.0076
G8E09_18170	DUF2938 domain-containing protein	-1.56	0.0001	0.0047
G8E09_10080	Acetoin reductase	1.56	0.0003	0.0119
G8E09_08695	CoA transferase subunit B	1.56	0.0001	0.0056
G8E09_13275	3-(3-hydroxy-phenyl)propionate transporter MhpT	1.56	0.0003	0.0119
G8E09_06835	3-hydroxyacyl-CoA dehydrogenase	1.56	0.0002	0.0083
G8E09_07815	Siderophore biosynthesis protein	-1.53	0.0005	0.0160
G8E09_09965	CoA transferase subunit A	1.52	0.0013	0.0324
G8E09_11795	MFS transporter	1.51	0.0000	0.0027
G8E09_06685	Transglutaminase family protein	1.51	0.0012	0.0307
G8E09_17135	DMT family transporter	1.50	0.0001	0.0048

*^*a*^log_*2*_ FC, log_*2*_ fold change; FDR, false discovery rate.*

**TABLE 4:** Differentially expressed genes in CAB010 compared with A1254.

**Gene_ID**	**Description**	**log2FC^*a*^**	***P*-value**	**FDR**
G8E09_16100	OXA-143 family carbapenem-hydrolyzing class D beta-lactamase OXA-499	2.04	0.0000	0.0014
G8E09_06800	LysR family transcriptional regulator	1.73	0.0000	0.0001
G8E09_06805	type 1 glutamine amidotransferase domain-containing protein	1.62	0.0000	0.0014

*^*a*^log_*2*_FC, log_*2*_ fold change; FDR, false discovery rate.*

## Discussion

*Acinetobacter pittii*, a member of the ACB complex, is increasingly recognized as a clinically important species following an improvement in identification methods that can better discriminate between *A. pittii* and *A. baumannii* (Yang et al., 2012). Here, we identified a carbapenem-non-resistant *A. pittii* clinical isolate, A1254, carrying *bla*_OXA–__499_, intrinsic *bla*_OXA–__826_, and *bla*_ADC–__221_. OXA-499 was first identified in a carbapenem-resistant *A. pittii* clinical isolate, YMC2010/8/T346, recovered from a patient in South Korea in 2010 and reported in 2017 (D’Souza et al., 2017). A1254 was also isolated in 2010. YMC2010/8/T346 is susceptible to imipenem (MIC, 2 μg/ml) but resistant to meropenem (MIC, 16 μg/ml). We compared the 4,085-bp *bla*_OXA–__499_ genetic environment between A1254 and YMC2010/8/T346 and found them to be identical. However, A1254 is susceptible to imipenem and intermediate-susceptible to meropenem based on the microbroth dilution method and even susceptible to meropenem when the Etest is used. The different MICs among *A. pittii* strains sharing an identical *bla*_OXA–__499_ genetic environment indicate that *bla*_OXA–__499_ expression might vary according to host and result in phenotypic variation, which might be due to the presence of additional related mechanisms that influence the phenotype.

However, when cloned into a vector, *bla*_OXA–__499_ containing either the initial or mutated promoter could confer carbapenem resistance in host strains. A similar result was reported by Zander et al. (2014a), who found that, although OXA-255 did not confer carbapenem resistance to the *A. pittii* clinical isolate AF726, OXA-255-transformed *A. baumannii* ATCC 17978 and *A. pittii* SH024 were resistant to carbapenem. Similar to OXA-499, OXA-255 is also a member of the OXA-143 family and the 2,239-bp genomic fragment containing *bla*_OXA–__255_ (GenBank accession number, KC479325) in *A. pittii* AF726 is similar to that of *bla*_OXA–__499_ in A1254 and YMC2010/8/T346 (99% identities). Considering that the genes were cloned into a shuttle vector and transformed into different *Acinetobacter* spp. strains by electroporation, we suspect that, apart from the genetic background of transformed strains, the introduction of the vector might also have influenced the expression level by providing multiple copies of the gene (dose effect), whereas in A1254, *bla*_OXA–__499_ was located on the chromosome and as a single copy.

RT-qPCR and RNA-Seq results (log_2_ fold changes) for *bla*_OXA–__499_ showed the same trend, although RNA-Seq data showed higher values. There was a significant difference in the number of differently expressed genes between CAB009 and CAB010 when compared with A1254. As CAB009 and CAB010 were isolated from two independently evolved populations, both mutants might harbor additional mutations besides the base substitutions in the promoter region of *bla*_OXA–__499_. The different genotypes of CAB009 and CAB010 might explain the differences in the transcriptional profiles.

Subinhibitory concentrations of antibiotics can lead to gene expression changes and promote resistance (Bernier and Surette, 2013; Chen et al., 2017). In this study, we demonstrated that a carbapenem-non-resistant strain, *A. pittii* A1254, which tends to be neglected in the clinical microbiology laboratory, can rapidly develop resistance to carbapenems when cultured in broth containing a low concentration of imipenem. Here, we evaluated the possible mechanisms underlying the increased expression level of *bla*_OXA–__499_ by analyzing the sequence of the promoter region. The mutant CAB009 isolate harbored an A to G transition at position −14, causing a base combination transformation from 5′-TA-3′ to 5′-TG-3′ one base upstream of the −10 region. The importance of the 5′-TG-3′ motif in the extended -10 element has been demonstrated in *E. coli* (Prost and Cozzone, 1999; Burr et al., 2000; Mitchell et al., 2003). It is well known that the −10 and −35 regions are where RNA polymerase (RNAP) contacts, resulting in promoter recognition and initiation of transcription. There is an additional promoter element, the “extended −10 element,” located one base upstream of the −10 region, with the major 5′-TG-3′ determinant positioned at −15/−14 with respect to the transcription start site (Mitchell et al., 2003). The 5′-TG-3′ motif is an important determinant of promoter activity (Voskuil and Chambliss, 2002; Prost and Cozzone, 1999; Burr et al., 2000). In our study, we suspect that it was the presence of the 5′-TG-3′ motif in the extended −10 element of the *bla*_OXA–__499_ promoter of the CAB009 mutant that led to the increased expression of *bla*_OXA–__499_, resulting in resistance to carbapenem. Another mutant, CAB010, exhibited a G to A transition at position −42, upstream of the −35 region. The UP element, located upstream of the −35 element (from approximately −40 to −60), can be recognized by RNAP and facilitates its initial binding as well as the subsequent steps in transcription initiation (Estrem et al., 1999; Presnell et al., 2019). A consensus UP element sequence consists almost exclusively of A and T residues and leads to increased promoter activity (Estrem et al., 1998). For the wild-type strain, *A. pittii* A1254, the proximal site of the UP element, contains a near-perfect A tract from position −39 to −44, interrupted only by a G at position −42. Following the transition from G to A at position −42, the proximal site in the CAB010 mutant became a perfect A tract.

In this study, we employed whole-genome sequencing (WGS) to identify putative resistance genes in the ancestor strain and mutations in the populations obtained from the experimental evolution. For clinical use, comprehensive databases of known resistant genes and related mutations are necessary for the successful prediction of antibiotic-resistance phenotypes. It is known that the accumulation of one or more single-nucleotide variants (SNVs) in genes encoding antibiotic targets or transposon insertions can lead to antibiotic resistance (Schurch and van Schaik, 2017). Published carbapenem resistance-related mechanisms in *A. pittii* include plasmid-borne *bla*_OXA–__23_, *bla*_OXA–__72_, or *bla*_OXA–__58_; AbaR4-located *bla*_OXA–__23_ on the chromosome; plasmid-borne class I integron containing *bla*_IMP–__1_ (Montealegre et al., 2012; Silva et al., 2018; Chen et al., 2019); and a composite transposon containing *bla*_NDM–__1_ (Yang et al., 2012). In addition, it has been proposed that *A. pittii* may be a resistance reservoir for the dissemination of NDM-1 (Bogaerts et al., 2013; Huang et al., 2015). Overexpression of *bla*_OXA_ is typically mediated through promoters provided by insertion sequence (IS) elements, although OXA-40 and OXA-143 appear to be exceptions to this (Higgins et al., 2009). The association with IS elements and frequent presence in plasmids highlight the potential of *bla*_OXA_ genes to spread within *Acinetobacter* spp. via transposition events and horizontal gene transfer (Zander et al., 2014b). For OXA-143-like and OXA-40-like, *bla*_OXA–__499_ is flanked by XerC/XerD-like recombinase sites in both *A. pittii* A1254 and YMC2010/8/T346, suggesting that this gene was acquired through recombination. This recombination system is exploited by mobile DNA elements to integrate into the host genome (Midonet and Barre, 2014). A similar genetic context was also reported for other *A. pittii* isolates, in which the resistance gene was flanked by XerC/XerD-like recombinase sites (Cayo et al., 2014; Ruan et al., 2017; Brasiliense et al., 2019). This suggests that *A. pittii* may be an important source of resistance genes and contribute to their dissemination among species.

Here, we report for the first time that mutations in the promoter region of *bla*_OXA–__499_ can contribute to the development of carbapenem resistance, complementing other known carbapenem resistance mechanisms in *A. pittii*. Enhanced resistance to β-lactam resulting from promoter mutations has also been reported in *Staphylococcus aureus* (Basuino et al., 2018). Additionally, loss of *pncA* expression due to promoter mutation conferred pyrazinamide resistance in multidrug-resistant tuberculosis isolates (Pang et al., 2017). This indicated that predicting resistance phenotypes based on the presence of resistance genes may be inaccurate in some circumstances; mutations on non-coding regions such as the promoter region should also be taken into consideration. One limitation of our study was that we failed to introduce a mutation in the wild-type A1254 strain due to technical restrictions. Moreover, we did not elucidate the specific mechanisms underlying the different carbapenem MICs between *A. pittii* strains harboring *bla*_OXA–__499_.

In conclusion, to the best of our knowledge, our study represents the first investigation on the development of carbapenem resistance in an OXA-499-harboring, but carbapenem-non-resistant, *A. pittii* isolate. The genetic environment of *bla*_OXA–__499_ was identical to that of a previously reported carbapenem-resistant *A. pittii* strain, indicating the existence of phenotypic variation in OXA-499-producing strains. We demonstrated that carbapenem-non-resistant *A. pittii* A1254 could become resistant to carbapenem under imipenem selective pressure and that a single-base substitution in the promoter region of *bla*_OXA–__499_ contributed to the carbapenem-resistance phenotype. This highlights the need to monitor the potential development of carbapenem resistance when treating infections caused by non-resistant strains. The potential risk of resistance development requires that more attention be paid to the type, courses, and doses of antibiotics prescribed.

## Data Availability Statement

The GenBank accession number of *bla*_OXA–826_ is MK810442 (https://www.ncbi.nlm.nih.gov/nuccore/MK810442). The GenBank accession number of *bla*_ADC–221_ is MN654470 (https://www.ncbi.nlm.nih.gov/nuccore/MN654470.1). The GenBank accession number of *A. pittii* A1254 is CP049806-CP049810 (BioProject: PRJNA610163). The SRA accession numbers for populations CAB001–CAB004 are SRR11306746, SRR11306745, SRR11306744, and SRR11306743, respectively (BioProject: PRJNA610163). The SRA accession numbers for raw reads of RNA-Seq are SRR11648396–SRR11648404 (BioProject: PRJNA610163).

## Author Contributions

YC, YY, and XHu designed the study. LZ, YF, XHa, and QX performed the experiments. XH, SW, BY, and LL analyzed the bioinformatics data. LZ and XHa wrote the manuscript.

## Conflict of Interest

The authors declare that the research was conducted in the absence of any commercial or financial relationships that could be construed as a potential conflict of interest.

## Footnotes

1 https://cge.cbs.dtu.dk/services/ResFinder/
