# Supplementary material for: Phenotypic Variation and Carbapenem Resistance Potential in OXA-499-Producing Acinetobacter pittii
Source: Front Microbiol. 2020 Jun 9;11:1134. doi: 10.3389/fmicb.2020.01134 (PMC7296048; doi:10.3389/fmicb.2020.01134)
Supplement: Supplementary file 1 [file Table_1.docx]

| population | accession number | position | mutation | frequency | annotation | locus_tag | description |
| --- | --- | --- | --- | --- | --- | --- | --- |
| CAB001 | CP049806  (chromosome) | 817180 | G→A | 100% | intergenic  (+343/‑126) | G8E09_03985→/→G8E09_03990 | sodium:proton antiporter/BapA prefix-like domain-containing protein |
| CAB001 | CP049806  (chromosome) | 831,329 | G→A | 77.70% | S4675N  (AGC→AAC) | G8E09_03990→ | BapA prefix-like domain-containing protein |
| CAB001 | CP049806  (chromosome) | 3,403,212 | A→G | 100% | intergenic  (‑200/‑44) | G8E09_16095←/→G8E09_16100 | TonB-dependent siderophore receptor/OXA-499 |
| CAB002 | CP049806  (chromosome) | 817,180 | G→A | 83.10% | intergenic (+343/‑126) | G8E09_03985→/→G8E09_03990 | sodium:proton antiporter/BapA prefix-like domain-containing protein |
| CAB002 | CP049806  (chromosome) | 831,329 | G→A | 86.90% | S4675N  (AGC→AAC) | G8E09_03990→ | BapA prefix-like domain-containing protein |
| CAB002 | CP049810  (plasmid) | 5173 | A→G | 94.00% | intergenic (+459/+23) | G8E09_19795→/←G8E09_19800 | helix-turn-helix domain-containing protein/hypothetical protein |
| CAB003 | CP049806  (chromosome) | 831,329 | G→A | 85.20% | S4675N  (AGC→AAC) | G8E09_03990→ | BapA prefix-like domain-containing protein |
| CAB003 | CP049810  (plasmid) | 5173 | A→G | 89.60% | intergenic (+459/+23) | G8E09_19795→/←G8E09_19800 | helix-turn-helix domain-containing protein/hypothetical protein |
| CAB004 | CP049806  (chromosome) | 831,329 | G→A | 100% | S4675N  (AGC→AAC) | G8E09_03990→ | BapA prefix-like domain-containing protein |

Table S1. Detected mutations with frequency greater than 70% in evolved populations

Table S2. Fold changes normalized by *rpoB* or *bla*_OXA-826_

| sample | ΔCT  (OXA499-rpoB) | fold change  (2^-ΔCT^) | ΔΔCT  (ΔCT-ΔCTA1254) | fold change  (2^-ΔΔCT^) |
| --- | --- | --- | --- | --- |
| A1254 | 1.44±0.23 | 0.37±0.06 | 0 | 1 |
| CAB009 | -3.09±0.06 | 8.51±0.38 | -4.53±0.19 | 23.18±3.03 |
| CAB010 | -0.21±0.47 | 1.2±0.42 | -1.65±0.25 | 3.16±0.57 |
| CAB009-IPM | -3.24±0.39 | 9.68±2.39 | -4.68±0.6 | 27.04±9.69 |
| CAB010-IPM | -1.10±0.38 | 2.2±0.6 | -2.54±0.15 | 5.83±0.62 |
| sample | ΔCT  (OXA826-rpoB) | fold change  (2^-ΔCT^) | ΔΔCT  (ΔCT-ΔCTA1254) | fold change  (2^-ΔΔCT^) |
| A1254 | 2.53±0.25 | 0.18±0.03 | 0 | 1 |
| CAB009 | 2.5±0.07 | 0.18±0.01 | -0.03±0.18 | 1.03±0.12 |
| CAB010 | 2.29±0.36 | 0.21±0.05 | -0.23±0.47 | 1.22±0.41 |
| CAB009-IPM | 2.34±0.34 | 0.2±0.05 | -0.18±0.49 | 1.18±0.43 |
| CAB010-IPM | 3.81±0.08 | 0.07±0.004 | 1.28±0.24 | 0.42±0.07 |
| sample | ΔCT(OXA499-OXA-826) | fold change  (2^-ΔCT^) | ΔΔCT  (ΔCT-ΔCTA1254) | fold change  (2^-ΔΔCT^) |
| A1254 | -1.09±0.35 | 2.17±0.52 | 0 | 1 |
| CAB009 | -5.58±0.12 | 48.1±3.94 | -4.49±0.24 | 22.74±3.62 |
| CAB010 | -2.50±0.12 | 5.68±0.47 | -1.41±0.23 | 2.69±0.44 |
| CAB009-IPM | -5.59±0.3 | 48.82±10.94 | -4.5±0.62 | 24.06±10.92 |
| CAB010-IPM | -4.90±0.46 | 31±10.39 | -3.81±0.28 | 14.24±2.58 |
